# Use of AI scribes in UK primary care: a survey of general practitioners

**DOI:** 10.1038/s41746-026-02762-8

**Published:** 2026-05-16

**Authors:** Christina Derksen, Mona Yekezare, Thomas Round, Suzanne E. Scott, Fiona M. Walter

**Affiliations:** 1 Wolfson Institute of Population Health, Faculty of Medicine and Dentistry, Queen Mary University of London, London, UK; 2 Barts and The London School of Medicine and Dentistry, Faculty of Medicine and Dentistry, Queen Mary University of London, London, UK; 3 School of Life Course and Population Sciences, King’s College London, London, UK

**Keywords:** Health care, Medical research, Risk factors

## Abstract

UK general practice is increasingly adopting ambient voice technology (“AI scribes”) for summarising and documenting medical consultations without clear regulation and implementation guidance. The aim of this study was to examine current use and perceptions of AI scribes among UK GPs, and to identify GP, practice, and population factors associated with their adoption. We conducted a cross-sectional online survey of UK GPs (*n* = 598) to examine current use, perceptions, and factors associated with adoption, analysed using logistic regression. AI scribes were used by 40% of GPs, with an additional 23% reporting past use. Use ranged from 5 to 100% of consultations (mean 60%). Adoption was more likely among men (OR = 1.64), GPs working in private practice (OR = 2.88) and 5–6 clinical sessions weekly (OR = 2.17), those with more experience (OR = 1.68), and GP trainers (OR = 3.10). Efficiency and timeliness were widely perceived benefits, while concerns about safety and medicolegal risks were common, particularly among non-users. Use of AI scribes in the UK is relatively high despite regulatory issues and recent official cease communication. Local population characteristics were not associated with use, but use varied significantly depending on GP characteristics. Selective use within practices and patient perspectives warrant further investigation to ensure equitable use of AI scribes.

## Introduction

Technological advances in Artificial Intelligence (AI) offer a range of opportunities to streamline and automate healthcare, including administrative tasks^1,2^. One example is ambient voice technology (AVT), also called ambient scribes or AI scribes. Unlike conventional dictation tools, AI scribes “listen” to consultations, capturing both the doctor’s and the patient’s speech. They transcribe and convert speech directly from the interaction into structured medical documentation, such as notes and letters^3^. Scaling the use of AI scribes is an explicit part of the National Health Service (NHS) 10-year health plan for England^4^.

Eccles et al. summarised potential positive and negative effects of AI scribes on six quality domains: safety, effectiveness, time, efficiency, equity, and patient-provider interaction^5^. In terms of benefits, recent systematic reviews, mainly including studies conducted in the United States (US), suggest that AI scribes can lessen administrative burden, decrease documentation time and increase documentation quality, reduce cognitive load and improve focus on the patient-provider interaction, leading to reduced burnout, higher patient satisfaction and potentially urgent access to care and conservation of healthcare budgets^6–8^. Primary care clinicians reported greater benefits than those in medical or surgical subspecialties^9^. Overall, studies measuring objective time spent on documentation in the Electronic Health Record (EHR) showed small benefits in time saved per patient, but up to 20 min of total time saved on EHR documentation per day^9–12^. However, there is high heterogeneity, with the 30% of top users accounting for 90% of AI scribe use^10^. Excessive length and the need for heavy editing increased general practitioner (GP) burden. Physicians in the US have also reported fears of being asked to see more patients due to reduced documentation efforts, as well as problems with AI scribe functionality and accuracy^13,14^.

Furthermore, clinicians reported inaccuracies such as mistaking “did” with “did not”, usability and integration issues and limited functionality for non-English speakers. If AI scribes are used selectively for English-speaking, health-literate patients, other patients who are already facing barriers to access and effective treatment might miss out on benefits. Additionally, larger or better-funded practices might be able to afford and implement AI scribes, which might widen performance gaps or reinforce geographic disparities, as shown by a recent Nuffield research report on use of AI in primary care, conducted in collaboration with the Royal College of General Practitioners^15^. Hence, AI scribes are posing a potential risk for patients or exacerbating inequalities^10,11^.

In the United Kingdom (UK), implementation of AI scribes in general practice differs from the US due to different EHR systems and information technology (IT) infrastructure, governance, and regulation^16^. UK implementation has been inconsistent, with NHS bodies highlighting potential benefits, encouraging use and providing guidance in April 2025^3,17,18^. However, NHS England also issued a cease letter in June 2025, asking GPs not to use AI scribes unless they are compliant with NHS standards around data management and patient safety, and reiterating that liability sits with individual users or practice^19^. Professional bodies have since taken a more cautious approach, advising use of AI scribes only if products have been registered as a medical device, and clinical safety and data protection assessments have been carried out^20^. It is difficult to estimate how many AI scribes are available for GPs in the UK. However, only a handful have proactively adhered to NHS standards^21–25^.

Despite the cease letter and more cautious approach, there are reports that GPs are using AI scribes that are not regulated, or do not consent patients adequately^26^. The recent Nuffield research report reported that 28% of 2108 survey respondents used AI tools in their practice, and that using AI scribes was the most common use case of AI^15^. This survey also highlighted that many AI tools were self-obtained rather than practice-supplied, leading to variation in AI adoption among GPs. Beyond this report, research on AI scribes in the UK is very scarce. A survey of 50 UK and Australian medical practitioners (published as an abstract in June 2025), reported that 28% were currently using AI scribes with mixed levels of satisfaction, and 48% were actively considering use^27^. In February 2024, 20% of 1006 GPs in the UK reported using any form of generative AI, of which 29% used AI for documentation purposes^28^. A follow-up survey in January 2025 showed that 69% of GPs believed AI could improve documentation^29^. However, this survey did not ask about AI scribes specifically.

In this fast-moving technological landscape, research is now needed to understand current use of AI scribes in the UK and how using AI scribes could affect primary care consultations and processes. Understanding potentially selective patterns of use could help to assess the risk of AI scribes in widening current inequalities. Hence, this research aims to explore: the proportion of UK GPs using AI scribes, and what systems are used; whether use varies by GP, practice, and local population characteristics; and GP perceptions of risks and benefits from using AI scribes in primary care.

## Results

### Participant and practice characteristics

600 GPs gave informed consent and completed the survey. Two were excluded due to conflicting responses, leaving *N* = 598 in the analyses. Many GPs were young (*N* = 433, 73%, under the age of 45), male (*N* = 344, 58%), and from diverse ethnic backgrounds (*N* = 386, 65%, from Asian, Black, Mixed or other backgrounds). GPs mostly worked as salaried GPs (*N* = 244, 41%), GP trainees (*N* = 139, 23%), or GP partners (*N* = 126, 21%), and between 3 and 8 clinical sessions per week (*N* = 529, 88%). Most worked in England (*N* = 527, 88%). Detailed characteristics and a comparison with the GP workforce in England are provided Table 1.

**Table 1:** GP and practice characteristics compared to the GP population in England (September 2025)

GP characteristics			*N* (%)	*N* (%) GP population in England^a^
Age		<34	167 (27.9)	11,492 (22.1)
		35–39	176 (29.4)	9648 (18.5)
		40–44	90 (15.1)	8411 (16.1)
		45–49	82 (13.7)	7407 (14.2)
		50–54	51 (8.5)	6102 (11.7)
		55–59	15 (2.5)	4398 (8.4)
		60+	11 (1.8)	4282 (8.2)
		Prefer not to say	6 (1.0)	368 (0.7)
Sex		Male	344 (57.5)	21,282 (40.8)
		Female	241 (40.3)	29,813 (57.2)
		Prefer not to say	13 (2.2)	1013 (1.9)
Ethnicity	Asian/Asian British	Indian	88 (14.7)	12,855 (25.7)
		Pakistani	60 (10.0)	
		Bangladeshi	9 (1.5)	
		Chinese	22 (3.7)	
		Other	16 (2.7)	
	Black/Black British	African	111 (18.6)	3505 (7.0)
		Caribbean	2 (0.3)	
		Other	6 (1.0)	
	Mixed or multiple	White and Asian	13 (2.2)	1265 (2.5)
		White and Black African	2 (0.3)	
		White and Black Caribbean	1 (0.2)	
		Other	9 (1.5)	
	White	English, Welsh, Scottish, Northern Irish or British	169 (28.3)	22,500 (44.9)
		Irish	5 (0.8)	
		Other	15 (2.5)	
	Other	Arab	41 (6.9)	1995 (4.0)
		Other	6 (1.0)	
		Prefer not to say	23 (3.8)	8605 (17.2)
Experience (years working as GP)		<1	54 (9.0)	
		1–5	217 (36.3)	
		6–10	132 (22.1)	
		11–15	81 (13.5)	
		16–20	53 (8.9)	
		21–25	41 (6.9)	
		26–30	15 (2.5)	
GP role		Salaried GP	244 (40.8)	20,552 (39.4)
		GP trainee	139 (23.2)	10,873 (20.9)
		GP partner	126 (21.1)	18,552 (35.6)
		Locum GP	69 (11.5)	1403 (2.7)
		Other	20 (3.3)	728 (1.4)
Clinical sessions per week		1–2	23 (3.8)	
		3–4	96 (16.1)	
		5–6	241 (40.3)	
		7–8	192 (32.1)	
		>9	46 (7.7)	
Private work		Yes, regularly	86 (14.4)	
		Yes, occasionally	136 (22.7)	
		No	376 (62.9)	
GP Trainer		Yes	196 (32.8)	
		No	402 (67.2)	
**Practice characteristics**				
IT system		EMIS Web	313 (52.3)	
		SystmOne TTP	240 (40.1)	
		Vision	27 (4.5)	
		Other (EPIC, Adastra, etc.)	19 (3.2)	
Practice region		North East England	26 (4.3)	8007 (15.4)
		North West England	73 (12.2)	
		Yorkshire & The Humber	36 (6.0)	6867 (13.2)
		West Midlands	58 (9.7)	9889 (18.9)
		East Midlands	90 (15.1)	
		East of England	48 (8.0)	5436 (10.4)
		Greater London	90 (15.1)	8067 (15.5)
		South East England	64 (10.7)	7286 (14.0)
		South West England	42 (7.0)	5541 (10.6)
		Scotland	24 (4.0)	
		Wales	19 (3.2)	
		Northern Ireland	15 (2.5)	
		n/a (unknown)	13 (2.2)	1025 (2.0)

^a^Taken from NHS Digital: https://digital.nhs.uk/data-and-information/publications/statistical/general-and-personal-medical-services. Ethnicity data was only available for broader categories.

### Use of AI scribes in primary care

Most GPs were either current users (*N* = 240, unweighted: 40%; weighted 43%) or past users of AI scribes (*N* = 236, unweighted: 23%; weighted: 21%). Of past users, two-thirds (*N* = 88/236, 65%) intended to use a scribe again in the future and almost one-third (*N* = 40/236, 29%) were unsure, with only 6% (*N* = 8/236) indicating they did not intend to use an AI scribe again. Among the never users (*N* = 222, unweighted: 37%; weighted: 37%), only 14% (*N* = 30/222) did not intend to use an AI scribe in the future.

Of the current users, over half (*N* = 131/240, 55%) had the AI scribe integrated into their computer system, compared to just over a quarter of past users (*N* = 38/236, 28%). Current users reported using AI scribes for an average of 60% (SD = 26.8; range 5–100%) of consultations, compared with 40% (SD = 26.2; range 1–100%) of consultations for past users.

Through conventional content analysis of the descriptions of AI use, a dominant category was *documentation and consultation support* (mentioned by current users: 185/216, 86%; past users: 100/125, 80%). GPs described using AI scribes primarily to streamline documentation during and after consultations to capture interactions with patients, taking patient history and summarising examination findings. Unstructured dictation was turned into clear and organised clinical notes. GPs reported using AI scribes especially for complex consultations, accurate and complete record keeping, and reducing the time and cognitive load associated with typing or structuring notes themselves. A second category centred around AI scribes *supporting communication with patients or secondary care* to reduce administrative burden and ensure clearer or more consistent communication across workflows. AI scribes were used to streamline tasks such as dictating or drafting letters, generating or completing referral documents, and sending messages or reminders to patients (mentioned by current users: 61/216, 28%; past users: 34/125, 27%). Some GPs described using AI scribes for *help with higher-level clinical decision-making* beyond intended use (current users: 20/216, 9%; past users: 6/125, 5%). The category reflects that GPs are using AI scribe functions beyond simple transcription and documentation. For example, GPs reported using AI scribes as support tools to interpret symptoms, refine diagnoses or identify possible differential diagnoses, therefore supporting their clinical reasoning. Other GPs reported using AI scribes to cross-check management plans, select interventions, and structure safety-netting, showing how AI scribes are integrated into the clinical management process. Finally, few GPs used AI scribes for *prescription support*, for example for repeat prescriptions or to facilitate prescribing more generally (current users: 8/216, 4%; past users: 1/125, 1%).

### Choice and reasons for discontinuation of AI scribes

GPs reported using 34 different AI scribes (Table 2). The most commonly used scribes were Heidi Health (current users: *N* = 128/240, 53%; past users: *N* = 77/136, 59%) and Accurx Scribe (current users: *N* = 77/240, 32%; past users: *N* = 35/136, 27%). Many never users were unsure which scribes they might use (*N* = 109/222, 49%) but those who knew commonly intended to use Heidi Health (*N* = 50/83, 60%) or Accurx Scribes (*N* = 45/83, 54%).

**Table 2:** AI scribes used by current and past users

	Current users (*N* = 240)		Past users (*N* = 136)	
Scribe	*N* (%^b^)	95% CI	*N* (%^b^)	95% CI
Heidi Health	128 (53.3)	46.8–59.7	77 (58.8)	49.8–67.2
Accurx Scribe	77 (32.1)	26.3–38.4	35 (26.7)	19.5–35.3
i-Scribe	18 (7.5)	4.6–11.8	5 (3.8)	1.4–9.1
Lexacom	16 (6.7)	4.0–10.8	9 (6.9)	3.4–13.0
CLEARNotes	15 (6.2)	3.7–10.3	4 (3.1)	1.0–8.1
Abridge	14 (5.8)	3.3–9.8	1 (0.8)	0.0–4.8
Anima	14 (5.8)	3.3–9.8	9 (6.9)	3.4–13.0
DAX Copilot	13 (5.4)	3.0–9.3	5 (3.8)	1.4–9.1
DeepScribe	13 (5.4)	3.0–9.3	5 (3.8)	1.4–9.1
HealthOrbit AI	13 (5.4)	3.0–9.3	4 (3.1)	1.0–8.1
Ambience Healthcare	11 (4.6)	2.4–8.3	1 (0.8)	0.0–4.8
Anthem	10 (4.2)	2.1–7.8	2 (1.5)	0.3–0.6
Other^a^	60 (25.0)	19.8–31.1	20 (15.3)	9.8–22.8

^a^Other AI scribes (used by less than 10 GPs per group) included: Lyrebird Health, Freed AI, Augmedix Go, Scribetech, Smart Notes AI dictation tool, BeamUp, OmniMD AI Scribe, Ezyscribe, TORTUS, Innovaccer Provider Copilot, Suki AI, TruBridge, Sunoh.ai, Twofold Health, Vero Scribe.

^b^Multiple answer options were possible, thus, the sum of percentages exceeds 100% per group.

Reasons for choosing AI scribes are presented in Table 3, with the most common reason for selection being a colleague’s recommendation.

**Table 3:** Reasons for choosing specific AI scribes

	Current users (*N* = 240)		Past users (*N* = 136)	
Reasons for choosing AI scribes	*N* (%^b^)	95% CI	*N* (%^b^)	95% CI
Colleague’s recommendation	151 (62.9)	56.4–69.0	95 (69.9)	61.3–77.3
Independent choice after hearing about it	49 (20.4)	15.6–26.2	32 (23.5)	16.9–31.7
Commissioned by the practice	63 (26.2)	20.9–32.4	17 (12.5)	7.7–19.5
Following advertisements on social media	31 (12.9)	9.1–18.0	14 (10.3)	6.0–17.0
Commission by ICB/health board	32 (13.3)	9.4–18.4	8 (5.9)	2.8–11.6
Vendor approaches	24 (10.0)	6.6–14.7	5 (3.7)	1.4–8.8
Other^a^	5 (2.1)	0.8–5.1	1 (0.7)	0.0–4.6

^a^Other reasons included recommendations by defence union, AI scribes popping up on toolbar, free trials, use in private practice.

^b^Multiple answer options were possible, thus, the sum of percentages exceeds 100% per group.

On average, past users had discontinued using an AI scribe just over 6 months previously (*M* = 6.6 months, SD = 9.1). Reasons for discontinuation are presented in Table 4 with the two most common reasons being lack of practice support and medicolegal and liability issues.

**Table 4:** Reasons for discontinuation of use of AI scribes

	Past users (*N* = 136)	
Reasons for discontinuation of AI scribes	*N* (%^b^)	95% CI
Lack of practice support for AI scribes	44 (32.4)	24.7–41.0
Medicolegal and liability issues	38 (27.9)	20.8–36.4
AI scribe no longer available	24 (17.6)	11.8–25.3
Concerns around the use of AI scribes	20 (14.7)	9.4–22.0
High costs	20 (14.7)	9.4–22.0
Lack of perceived benefits	13 (9.6)	5.4–16.1
Official communication asking to cease use	12 (8.8)	4.8–15.2
Negative media reports	5 (3.7)	1.4–8.8
Other^a^	17 (12.5)	7.7–19.5

^a^Other reasons included waiting for new AI scribe, lack of integration into system, recent change in workplace, unsure if approved by integrated care system, too expensive for locum GP, practice decision to pause use of AI scribes, end of trial, personal preference, and broken computer microphone.

^b^Multiple answer options were possible, thus, the sum of percentages exceeds 100% per group.

### Factors associated with the use of AI scribes

Univariate logistic regression analyses (see Table 5) indicated that men were more likely to use AI scribes than women (OR = 1.64 [95% CI: 1.17–2.31]). GPs with more experience were more likely to use AI scribes than those with 5 years or less (6–15 years: OR = 1.68 [95% CI: 1.16–2.45]; 16+ years: OR = 1.63 [95% CI: 1.03–2.62]), and those working 5 or more clinical sessions per week were more than twice as likely to use AI scribes than those working fewer sessions (5–6 sessions: OR = 2.17 [95% CI: 1.39–3.40]; 7+ clinical sessions: OR = 2.83 [95% CI: 1.80–4.48]). GP trainers were approximately three times more likely to use AI scribes (OR = 3.10 [95% CI: 2.10–4.65]), and those working in private practice regularly (OR = 2.88 [95% CI: 1.69–5.10]) or occasionally (OR = 2.45 [95% CI: 1.59–3.84]) were more than twice as likely to use AI scribes than those who did not. GP trainees (OR = 0.48 [95% CI: 0.29–0.79]) and those in other roles (e.g., retainer/portfolio GPs, OR = 0.11 [95% CI: 0.03–0.32]) were much less likely to use AI scribes than GP partners, but numbers were low in the latter group. Ethnicity and IT system were not associated with use of AI scribes. Finally, GPs working in regions with higher proportions of disability in the community were slightly less likely to use AI scribes (OR = 0.94 [95% CI: 0.89–0.99]), but other local authority characteristics were not associated with use of AI scribes.

**Table 5:** Univariate regression analysis on use of AI scribes (ever use vs never use)

		Ever users *n* (%)	Never users *n* (%)	Estimate	S.E.	*p*-value	OR	95% C.I. for Odds ratio	
								Lower	Upper
Age (in years)	**<39**	213 (62.1)	130 (37.9)	1					
	40–49	113 (65.7)	59 (34.3)	0.16	0.20	0.424	1.17	0.80	1.72
	50+	47 (61.0)	30 (39.0)	−0.04	0.26	0.863	0.96	0.58	1.60
Sex	**Women**	136 (56.4)	105 (43.6)	1					
	Men	234 (68.0)	110 (32.0)	0.50	0.17	0.004	1.64	1.17	2.31
Ethnicity	**White**	118 (59.6)	80 (40.4)	1					
	Asian or Asian British	125 (64.1)	70 (35.9)	0.19	0.21	0.358	1.21	0.81	1.82
	Black or Black British	81 (68.1)	38 (31.9)	0.37	0.24	0.132	1.45	0.90	2.35
	Mixed or multiple ethnic groups	11 (68.8)	5 (31.3)	0.40	0.56	0.474	1.49	0.52	4.88
	Other	30 (63.8)	17 (36.2)	0.18	0.34	0.594	1.20	0.63	2.35
Experience(in years)	**<5**	156 (56.5)	120 (43.5)	1					
	6–15	146 (68.5)	67 (31.5)	0.52	0.19	0.007	1.68	1.16	2.45
	16+	74 (67.9)	35 (32.1)	0.49	0.24	0.041	1.63	1.03	2.62
Current role	**GP Partner**	88 (69.8)	38 (30.2)	1					
	Salaried GP	168 (68.9)	76 (31.1)	-0.05	0.24	0.845	0.96	0.60	1.52
	GP trainee	73 (52.5)	66 (47.5)	-0.74	0.26	0.004	0.48	0.29	0.79
	Locum GP	43 (62.3)	26 (37.7)	-0.34	0.32	0.286	0.71	0.39	1.33
	Other	4 (20.0)	16 (80.0)	-2.23	0.59	<0.001	0.11	0.03	0.32
Clinical sessions	**1–4**	54 (45.4)	65 (54.6)	1					
	5–6	167 (70.2)	71 (29.8)	0.77	0.23	<0.001	2.17	1.39	3.40
	7+	155 (64.3)	86 (35.7)	1.04	0.23	<0.001	2.83	1.80	4.48
Private work	**No private work**	207 (55.1)	169 (44.9)	1					
	Occasional	102 (75.0)	34 (25)	0.90	0.22	<0.001	2.45	1.59	3.84
	Regular	67 (77.9)	19 (22.1)	1.06	0.28	<0.001	2.88	1.69	5.10
GP trainer	**No**	221 (55.0)	181 (45.0)	1					
	Yes	155 (79.1)	41 (20.9)	1.13	0.20	<0.001	3.10	2.10	4.65
IT system	**EMIS Web**	190 (60.9)	122 (39.1)	1					
	SystmOne TTP	163 (67.9)	77 (32.1)	0.31	0.18	0.089	1.36	0.96	1.94
	Other	23 (50.0)	23 (50.0)	−0.44	0.32	0.162	0.64	0.34	1.20
Population density				−0.00	0.00	0.538	1.00	0.99	1.00
Household language				0.01	0.01	0.349	1.01	0.99	1.03
Population age				0.03	0.03	0.361	1.03	0.97	1.09
Population ethnicity				0.00	0.01	0.655	1.00	0.99	1.01
Economic activity				−0.01	0.02	0.703	0.99	0.95	1.03
Education				−0.01	0.01	0.291	0.99	0.97	1.01
Disability				−0.06	0.03	0.021	0.94	0.89	0.99

The multivariable logistic regression (see Supplementary Table 1) showed similar results, with the additional finding that GPs working with SystmOne TTP were more likely to use AI scribes than those working with EMIS Web (OR = 1.65 [95% CI: 1.10–2.48]), and that experience working as a GP and being a trainee were no longer associated with using AI scribes.

### Perceived benefits and risks of AI scribes

High proportions of GPs agreed there would be benefits of using AI scribes for GPs (69–96%), patients (64–84%), the GP practice (71–94%), and the NHS (73–91%), with a higher proportion of current users agreeing with benefits than past or never users. Fewer GPs agreed there were risks of using AI scribes, although over 50% agreed there would be risks to GPs, and over 40% agreed there would be risks for patients, the GP practice and the NHS (see Supplementary Table 2 for more details).

**Fig. 1:**
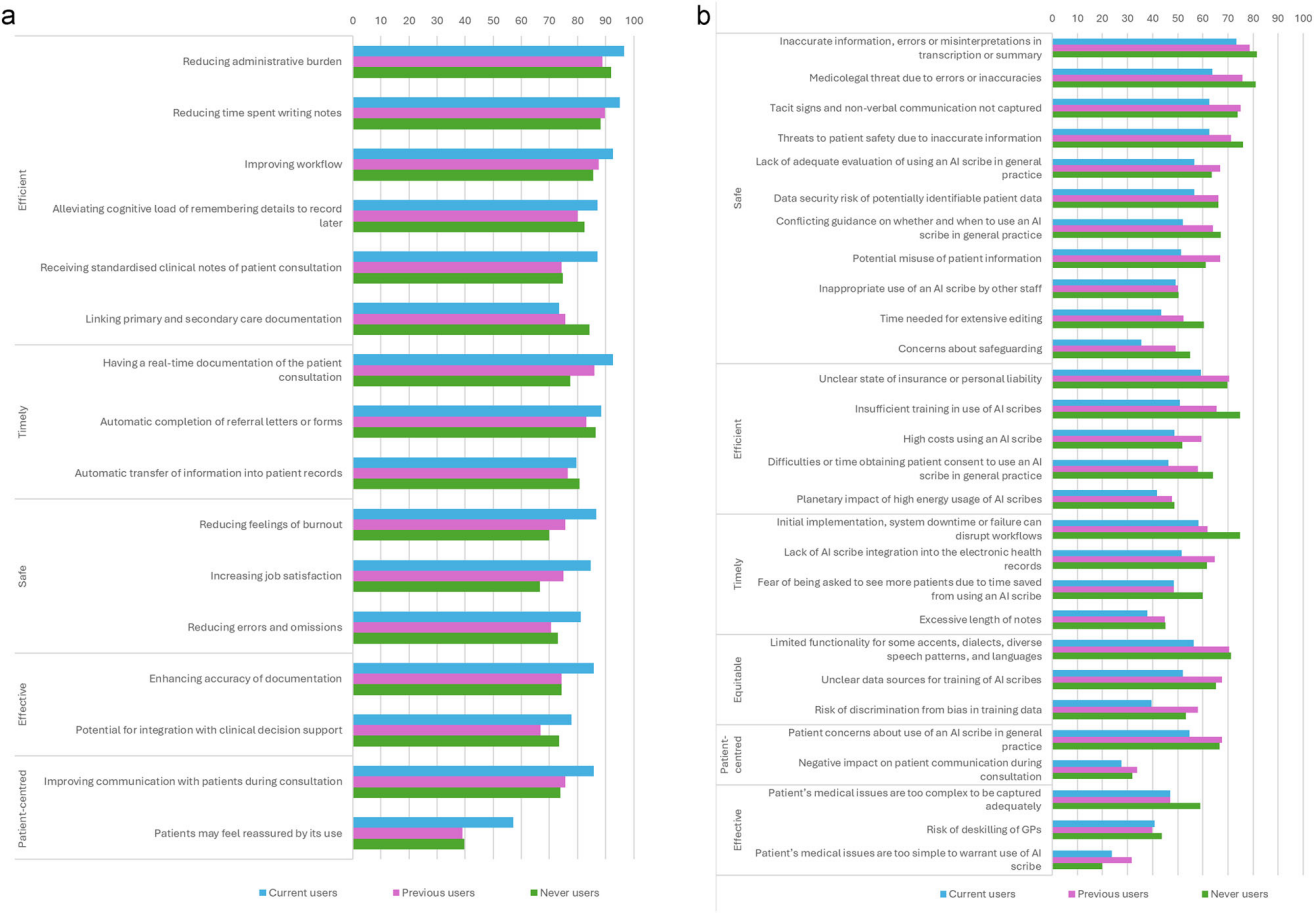
Endorsement of benefits and risks of AI scribes by user group. **a** Percentage of current users, past users and never users endorsing the benefits of using AI scribes. **b** Percentage of current users, past users and never users endorsing risks of using AI scribes.

The specific benefits of AI scribes were endorsed by most participants, regardless of current use. Over 75% endorsed the benefits associated with timeliness, such as having real-time documentation, over 70% endorsed the benefits associated with efficiency (e.g., reducing administrative burden; time spent writing notes) and over 65% endorsed benefits associated with safety (e.g., reducing feelings of burnout; reducing errors and omissions). However, only 40% of past users and never users agreed that use of AI scribes would reassure patients (Fig. 1a and Supplementary Table 2).

A substantial proportion of GPs endorsed the specific risks of AI scribes, particularly those associated with safety (e.g., over 60% agreed there are risks of inaccuracies, errors, misinterpretations and associated medicolegal threats), timeliness (e.g., over 50% agreed there are risks of disruption to workflow and lack of integration to electronic health records), and risk of inequity including limited functionality for some patient groups. Past users and never users were descriptively more likely to endorse risks than current users (Fig. 1b). Content analysis resulted in few additional specific benefits, such as support for GPs with specific needs (e.g., neurodivergence or wrist injuries), and risks such as AI scribes being disruptive to shared decision making and the threat of staff being made redundant if AI adoption saves administrative time.

## Discussion

This survey indicates that approximately 40% of UK GPs are currently using AI scribes. Adoption was largely informal, driven by peer suggestion rather than systematic rollout. GPs mostly used AI scribes for administration and documentation, or to support communication with patients and secondary care. Over 20% of GPs had discontinued use, mainly due to limited practice support and medicolegal concerns. Usage was associated more with GP and practice characteristics than local population factors, suggesting that adoption may not exacerbate inequalities geographically, but potentially within practices. Benefits regarding efficiency, timeliness, and reduction of burnout were endorsed by most GPs, but concerns around safety and medicolegal risks persist.

Existing literature on AI scribes is dominated by policy and expert commentary^5,30,31^ with limited primary research on current use and implications for primary care. Our survey is among the first UK studies to assess adoption patterns of AI scribes rather than use of AI in general, across a large and diverse GP sample in the UK. We piloted the survey with practicing GPs, albeit a small convenience sample. However, the self-reported survey could not objectively measure actual usage patterns, and use of AI scribes seems to change rapidly. Recruitment via a medical research company led to overrepresentation of younger, male, and Black GPs compared to the UK’s GP workforce. We used survey weighting to partially account for this and found small differences; however, non-probabilistic recruitment and low weighting efficiency required weighting schemes to be adjusted. Household language data were unavailable for Scottish local authorities, reducing data available for these variables in the regression analyses. Finally, the qualitative data were limited to short answers to open-ended questions to assess what GPs used AI scribes for, and whether they perceived risks and benefits beyond the comprehensive item lists we provided. This approach limited the richness of the qualitative analyses, and future research should consider qualitative approaches to investigate perceived benefits and barriers to AI scribe use in more depth.

The findings support and enhance the recent Nuffield report, echoing findings of variable adoption of AI among GPs, and concerns about use^15^. We have added important insights, by specifically focusing on AI scribes, and looking at past use as well as current use. In line with research from the UK and further afield^27,28,32^ the findings show that, despite caution from professional bodies^19,20^, AI scribes are increasingly common in primary care. However, adoption even within users is variable and ranges from 5 to 100% of consultations.

GPs endorsed benefits and risks similar to those identified in US studies^8–12^ and policy analyses^5^ and highlighted additional benefits, such as support for neurodivergent clinicians or those with typing difficulties. Some benefits might be related to practitioner characteristics. For instance, time saved on documentation and reduced feelings of burnout are likely to be more relevant to GPs working more clinical sessions^10,11^. Practical or medicolegal concerns may also be more difficult to overcome for some GPs. For example, it might be easier for GP partners than trainees to implement AI scribes in their practices. GP trainees were only half as likely to use AI scribes. Concerns remained that efficiency gains could paradoxically lead to redundancies or increase workload if GPs were required to see more patients^8^. Perceived risks were more likely to be endorsed by never users and should be targeted for broader acceptance of AI scribes.

Encouragingly, population characteristics were not associated with adoption of AI scribes, but disparities emerged by GP and practice factors. This is in contrast to the Nuffield finding that reported higher use of AI by GPs who worked in more affluent areas (self-reported rather than using index of multiple deprivation as in the current study)^15^. Male GPs were more likely to use AI scribes, consistent with prior findings of more positive attitudes towards AI among men^15,29^. GPs conducting more private work were two to three times more likely to use AI scribes, potentially due to fewer barriers in access to technology, financial constraints, or regulatory expectations^33,34^. More experienced GPs and especially GP trainers were also more likely to be users, suggesting that awareness of new innovations and resource availability facilitates use of AI scribes, despite common narratives that younger GPs take up new technology faster.

Practice-level support and IT resources are likely affecting adoption and discontinuation. This highlights the current responsibility of individual practices in commissioning and governing AI scribes, which is complicated by uncertainty around medicolegal practice, changing availability of AI scribes, and technical barriers such as poor integration with existing systems^35^.

Variation in use, reliance on peer recommendations, and safety concerns suggest rapid but uncontrolled integration into primary care, which could exclude patients already experiencing barriers to accessing primary care and effective communication from experiencing AI scribe benefits^15^. Evidence-based national guidance is required to address variation and inconsistent implementation^15,29^. Professional standards addressing liability, data security, training, and clinical use are needed, alongside feedback loops to detect problems post-implementation.

Given pressures on primary care and rising costs of technology, it is essential to understand potential health economic and productivity benefits of AI scribes. The NHS 10-year plan^4^ encourages community-based, digital care, including AI, but support is needed for commissioning, funding, and implementation. Without this, uncontrolled roll-out remains a risk, with practices potentially having unlimited liability for any data errors or breaches.

Ambient voice technology is one of several AI innovations targeting administrative burdens in healthcare^4^. However, some GPs in our study reported using AI scribes beyond documentation, including decision-making and communication with secondary care. Replacing GPs’ clinical reasoning with AI scribe functions could have serious implications for patient safety^5,36^. Clinical notes do not only function as records of consultations, but the process of taking notes supports clinical reasoning and decision-making^37^. Our qualitative results indicate that delegating note-taking to AI tools might interfere with decision-making processes, but there is scope for AI scribes to support higher-level reasoning. Further mixed-methods research, including qualitative, observational, implementation science, and health economic studies, should evaluate the effects of AI scribes on clinical reasoning processes and integration with primary care workflows, and assess cost-effectiveness.

Previous research has found mixed patient attitudes towards AI technology^38^, which was mirrored by uncertainties reported by GPs in this study. Future work should explore patient perspectives, clarify consent requirements and processes, and assess differential impacts. While population-level biases were limited, higher use in private practice raises equity concerns due to selective barriers to accessing private healthcare.

AI scribes are increasingly embedded in UK primary care, and use varies by GP and practice characteristics. Reported benefits include efficiency, improved documentation, enhanced patient engagement, and emerging use of AI scribes in communication and decision-making. Concerns remain around patient safety, medicolegal liability, equity in realising benefits equally, and governance. In the absence of standardised guidance, there is an urgent need for national guidelines, evaluation of accuracy and safety, and coordinated implementation to ensure AI scribes strengthen rather than undermine the quality of efficient and safe primary care.

## Methods

### Study design

We conducted a cross-sectional online survey, adapted from a similar survey on availability and use of electronic Clinical Decision Support (eCDS)^39^. This instrument was chosen as both eCDS and AI scribes function as digital clinical tools supporting documentation and decision-making. Items captured constructs that were relevant to the aims of this survey, including availability and use in clinical practice. Ethical approval for this study was received from Queen Mary Ethics of Research Committee (reference QME25.1282). All participants gave informed consent.

### Sample

Between 11th September and 7th October 2025, we recruited currently practising GPs in the UK via the medical market research company Sermo. Recruitment via a market research company enabled fast access to a large, verified, and diverse pool of practising GPs in the UK. GPs were reimbursed £20 in line with the company guidance. Recruitment was not monitored regarding quotas until *N* = 500 GPs were recruited. At this point, GPs were predominantly under the age of 45 years, so we implemented quotas to recruit an extra *N* = 100 GPs above this age group.

### Procedure

The survey, hosted on Qualtrics, asked about GP characteristics, practice characteristics, use of AI scribes and perceived risks and benefits of AI scribes, after a linked participant information sheet and consent form (see Supplementary Survey). We piloted the survey in Think Aloud sessions^40^ with a small convenience sample (*N* = 5) of GPs. In response, minor adjustments were made to improve clarity.

#### GP characteristics

Measures of GP characteristics included experience (in categories of 5 years), current role (GP partner, salaried GP, locum GP or GP trainee), GP trainer (yes/no), number of clinical sessions per week (1–2, 3–4, 5–6, 7–9, >9), private medical work (regularly/occasionally), age (in categories of 5 years), sex, gender, and ethnic group (using wording from the Office of National Statistics 2021 Census for England and Wales^41^). Clinical sessions refer to approximately 4 h of clinical activity, including consultations, admin, results handling, supervision, and related clinical tasks. Two sessions make up a typical working day.

#### Practice characteristics

Practice characteristics included the IT system and local authority. This was used to derive practice region and link to population data provided by the Office of National Statistics (ONS^42^), National Records of Scotland (NRS^43^) and Northern Ireland Statistics and Research Agency (NISRA^44^). Population variables retrieved were density (people/km²), household language (percentage of households with non-English adults, not available for Scotland), mean age, ethnicity (combined percentage of Asian/Asian British, Black/Black British, Mixed/Multiple groups), economic activity (percentage economically inactive), education (percentage level 3 or lower qualification), and disability (percentage mild or severe). Where local authority data were unavailable, MSOA data from ONS were aggregated.

#### Use of AI scribes

GPs were asked about their use of AI scribes in consultations, with response options of current, past, or never use. Current users and past users were asked to name the AI scribes they used from an alphabetical list, including those known to be registered as class one medical devices or piloted in primary care. GPs were asked how they selected the scribe, when they first used it (month/year since January 2020), the proportion of consultations in which it was applied, and its purposes (open-answer format; “What are you using an AI scribe for?”). Past users were also asked when and why they stopped (multiple-choice question with an option to specify further). Never users were asked to indicate whether they intended to adopt AI scribes in the future, and which scribe(s).

#### Benefits and risks of AI scribes

Drawing on published US research and UK policy literature^5–7,9–14^, GPs were asked about perceived benefits and risks of AI scribes. First, they rated agreement on a 5-point Likert scale (from “strongly disagree” to “strongly agree”) about whether AI scribes had benefits or risks for different stakeholders: themselves as GPs, patients, their practice, and the NHS. Second, they responded to specific statements (e.g., “Time needed for extensive editing” with a binary answer format (yes/no)), followed by open-ended text fields for further comments.

### Analysis

Quantitative analyses were conducted in R (version 4.3.1). As mandatory responses minimised missing data, no imputation was applied. Data from consenting GPs, analysis plan, and analysis code are available via OSF: https://osf.io/2bre9/files.

Participant and practice characteristics are reported as numbers and percentages relative to the full sample.

Descriptive statistics on the use of AI scribes are reported as proportions with 95% confidence intervals (CI). Frequencies of AI scribe use, but no other variables, were weighted using iterative proportional fitting (raking) to align the sample with the GP workforce in England^45^ by sex (men, women, unknown) and role (Partner, Salaried, Trainee, Locum, other). Effective sample size (*N*_eff_=443, ratio 74.2) and design effects (*D*_eff_=1.35) were calculated. Univariate and multivariable logistic regression examined associations between GP/practice characteristics and AI scribe use (ever vs never). Variables with *p* < 0.20 in univariate analyses were included in multivariable models to avoid excluding potentially relevant factors. Population density was recoded per 100 persons/km² to yield interpretable Odds Ratios and CIs^46^.

Types of AI scribes for current and past users, reasons for choosing specific AI scribes for current and past users, and reasons for discontinuation for past users, are reported as proportions with 95% confidence intervals (CI).

Perceived benefits and risks were reported by frequency and grouped into quality domains according to Eccles et al.^5^.

To further understand whether GPs used AI scribes as intended or for additional purposes beyond documentation, as well as to capture additional perceived risks and benefits, open responses were analysed using a conventional content analysis^47^. As survey answers were often short (e.g., “Note taking, letter generation”, “Automatic completion of referral letters”), inductive codes are descriptive rather than interpretive. First, one author (MY) repeatedly read all responses and inductively assigned codes to summarise responses. Codes were iteratively refined and grouped into broader categories. Answers could be coded into more than one category if they represented different aspects of use or different benefits or risks. A second author (CD) independently reviewed a subset of responses to check clarity, consistency, and alignment with the developed coding frame. The full author team reviewed and agreed the final coding structure. The number of GPs mentioning certain categories are used to illustrate the proportion of GPs using AI scribes as intended for support with documentation during consultations or for functions beyond simple documentation support. Although the frequencies of open-ended answers cannot be seen as representative for the sample, they reflect what are more or less common uses of AI scribes.

#### Sample size

To estimate AI scribe use among UK GPs (≈54,000), a sample of 384 was required to estimate proportions with 5% margin of error and 95% confidence. To investigate factors associated with the use of AI scribes using logistic regression, assuming an odds ratio of 1.89 (reflecting a 0.15–0.25 difference in likelihood based on prior literature^27^ and clinical input), a sample of 505 was needed to achieve 80% power at *α* = 0.05.

### Patient and public involvement (PPI)

Two PPI representatives reviewed the survey from a patient perspective, suggesting clearer wording and greater emphasis on benefits and risks, particularly through open-answer options. One raised concern about the representativeness of the sample, noting the survey might attract GPs supportive of AI scribes. Findings were shared and discussed with the representatives, including implications for future research and practice. Discussions covered lay-friendly communication of risks and benefits, medicolegal requirements, patient consent, and feedback loops to address accuracy issues after implementation.

## Supplementary information

AI scribes suppl_revision.

## Data Availability

Data from consenting GPs are available via OSF: https:/osf.io/2bre9/files .

## Code availability

The analysis code is available via OSF: https://osf.io/2bre9/files.
