# Supplementary material for: Use of AI scribes in UK primary care: a survey of general practitioners
Source: NPJ Digit Med. 2026 May 16;9:596. doi: 10.1038/s41746-026-02762-8 (PMC13433917; doi:10.1038/s41746-026-02762-8)
Supplement: Supplementary file 1 [file 41746_2026_2762_MOESM1_ESM.docx]

# Supplementary materials 1: Survey

## Start page

Dear GP,

Thank you for taking the time to share your views about the current use of AI scribes in primary care/ general practice. AI scribes are software applications that use Artificial Intelligence to automate clinical documentation, mostly by transcribing and summarising doctor-patient consultations in real time. They are also called ‘ambient scribes’ and include advanced ‘ambient voice technologies’ (AVTs) and are based on Large Language Models (LLMs).

We wish to understand how many GPs from across the UK have AI scribes available and are using them, whether use varies by GP and patient population characteristics, and the perceived risks and benefits of AI scribes. The survey will take approximately 5-10 minutes.

You can find more information here: [Participant Information Sheet](https://wiphbehaviouralsci.qualtrics.com/CP/File.php?F=F_54JcwukqCEkYNsq).

If you have any questions, please contact Dr Christina Derksen, Postdoctoral Research Associate, Queen Mary University of London.
Email: [christina.derksen@qmul.ac.uk](mailto:christina.derksen@qmul.ac.uk?subject=AI%20scribes%20survey)
Phone: 07586 763123.


Before you start the survey, please carefully consider the statements below.

## Consent

| **Statement** | **I agree** | **I don’t agree**^1^ |
| --- | --- | --- |
| 1. I confirm that I have read the Participant Information Sheet linked above. I have had the opportunity to consider the information, ask questions via email/ telephone and have had these answered satisfactorily. |  |  |
| 2. I understand that my participation is voluntary and that I am free to stop taking part in the study at any time without giving any reason and without my rights being affected. |  |  |
| 3. I understand that my anonymous data will only be accessed by the research team (researchers and students working with Dr Christina Derksen, Professor Suzanne Scott, and Professor Fiona Walter). |  |  |
| 4. I understand that my anonymous data will be securely stored in a secure electronic folder at Queen Mary University of London and in accordance with the data protection guidelines of the Queen Mary University of London for five years after the end of the project in fully anonymised form. |  |  |
| 5. I understand that because the data is processed anonymously, I will not be able to request withdrawal of the information I have provided after completing this survey. |  |  |
| 6. I agree that quotes from my answers in text boxes written in my own words are published as part of this research in anonymous form (optional). |  |  |
| 7. I understand that the researcher cannot identify me in any publications and other study outputs, as no identifiable information is obtained from this study. |  |  |
| 8. I understand that the information collected about me might be used to support other research in the future, and it may be shared in anonymous form with other researchers (optional). |  |  |
| 9. I agree to take part in this study. |  |  |

*Note: ^1^If participants selected “I don’t agree” for any question except for questions 6 and 8, they were automatically redirected and excluded from the study.*

## GP and practice characteristics

Questions in this section will allow us to understand how views differ between different people and whether our sample is representative of GPs in the UK.

1. Which clinical computer system do you use in your general practice?

- EMIS Web
- SystmOne TPP
- Vision
- Other, please specify: __________.

2. How many years have you practiced as a GP?

- <1
- 1-5
- 6-10
- 11-15
- 16-20
- 21-25
- 26-30
- >30

3. What is your position in your current practice?

- GP partner
- Salaried GP
- Locum GP
- GP trainee
- Retired or not practicing [screened out]
- Other, please specify: _________.

4. How many clinical sessions in general practice do you work per week?

- 1-2
- 3-4
- 5-6
- 7-8
- >9

5. In the past 12 months, have you undertaken any private medical work in addition to your NHS role?

- Yes, on a regular basis
- Yes, on an occasional basis
- No

6. Are you a qualified GP trainer for GP trainees?

- Yes
- No

7. How old are you (in years)?

- <34
- 35-39
- 40-44
- 45-49
- 50-54
- 55-59
- 60+
- Prefer not to say

8. What is your sex?

- Female
- Male
- Prefer not to say

9. Is the gender you identify with the same as your sex registered at birth?

- Yes
- No, enter gender identity: __________
- Prefer not to say

10. What is your ethnic group?

**White**

- English, Welsh, Scottish, Northern Irish or British
- Irish
- Gypsy or Irish Traveller
- Roma
- Any other White background, please describe: __________

**Mixed or Multiple ethnic groups**

- White and Black Caribbean
- White and Black African
- White and Asian
- Any other Mixed or Multiple ethnic background, please describe: __________

**Asian or Asian British**

- Indian
- Pakistani
- Bangladeshi
- Chinese
- Any other Asian background, please describe: __________

**Black, Black British, Caribbean or African**

- African
- Caribbean
- Any other Black, Black British, Caribbean or African background, please describe: __________

**Other ethnic group**

- Arab
- Any other ethnic group, please describe: __________
- Prefer not to say

11. Please provide your local authority. This will enable us to characterise your patient population without identifying you or your practice. Please start typing to enable the search function.

[Drop down menu country/ local authority with “I don’t know” option]

12. If you cannot find your local authority above, please write it in the text box below:

[Open text box]

## Your experience of AI scribes

13. Are you currently using an AI scribe in your consultations in general practice?

- Yes, I am currently using an AI scribe.
- No, but I have previously used an AI scribe.
- No, I have never used an AI scribe.

Blocks for

- Current users (option 1 above)
- Previous users (option 2 above)
- Never users (option 3 above)

## Current user block

14. Is the AI scribe integrated into your computer system?

- Yes
- No
- I don’t know

15. Which AI scribe(s) do you currently us in general practice? Please select all that apply.

- Abridge
- Accurx Scribe (Tandem Health)
- Ambience Healthcare
- Anima
- Anthem
- Augmedix Go
- BeamUp
- CLEARNotes
- DAX Copilot (Microsoft)
- DeepScribe
- Ezyscribe (Pennhealth Informatics)
- Freed AI
- HealthOrbit AI
- Heidi Health
- Innovaccer Provider Copilot
- i-Scribe
- Lexacom
- Lyrebird Health
- OmniMD AI Scribe
- Scribetech (Augnito Omni AI)
- Smart Notes AI dictation tool (The Access Group)
- Suki AI
- Sunoh.ai
- TORTUS
- TruBridge
- Twofold Health
- Vero Scribe
- Other, please specify: __________

16. Which AI scribe(s) have you previously used in general practice? Please select all that apply.

- I have not previously used another AI scribe.
- Abridge
- Accurx Scribe (Tandem Health)
- Ambience Healthcare
- Anima
- Anthem
- Augmedix Go
- BeamUp
- CLEARNotes
- DAX Copilot (Microsoft)
- DeepScribe
- Ezyscribe (Pennhealth Informatics)
- Freed AI
- HealthOrbit AI
- Heidi Health
- Innovaccer Provider Copilot
- i-Scribe
- Lexacom
- Lyrebird Health
- OmniMD AI Scribe
- Scribetech (Augnito Omni AI)
- Smart Notes AI dictation tool (The Access Group)
- Suki AI
- Sunoh.ai
- TORTUS
- TruBridge
- Twofold Health
- Vero Scribe
- Other, please specify: __________

17. How did you choose the AI scribe? Please select all that apply.

- A colleague recommended it to me.
- I decided to use it independently after hearing about it.
- I saw an advertisement on social media.
- A vendor approached me/ my practice.
- It was commissioned by my practice.
- It was commissioned for my practice by our ICB/ health board.
- Other, please specify: __________.

18. When did you first use an AI scribe in general practice?

[Dropdown month/ year with “I don’t know” option for month]

19. For what proportion of consultations do you currently use an AI scribe in general practice? Please estimate.

[slider 0-100%]

20. What are you using an AI scribe for?

[Open text box]

## Previous user block

14. Was the AI scribe integrated into your computer system?

- Yes
- No
- I don’t know

15. Which AI scribe(s) did you previously use in general practice? Please select all that apply.

- Abridge
- Accurx Scribe (Tandem Health)
- Ambience Healthcare
- Anima
- Anthem
- Augmedix Go
- BeamUp
- CLEARNotes
- DAX Copilot (Microsoft)
- DeepScribe
- Ezyscribe (Pennhealth Informatics)
- Freed AI
- HealthOrbit AI
- Heidi Health
- Innovaccer Provider Copilot
- i-Scribe
- Lexacom
- Lyrebird Health
- OmniMD AI Scribe
- Scribetech (Augnito Omni AI)
- Smart Notes AI dictation tool (The Access Group)
- Suki AI
- Sunoh.ai
- TORTUS
- TruBridge
- Twofold Health
- Vero Scribe
- Other, please specify: __________

16. How did you choose the AI scribe? Please select all that apply.

- A colleague recommended it to me.
- I decided to use it independently after hearing about it.
- I saw an advertisement on social media.
- A vendor approached me/ my practice.
- It was commissioned by my practice.
- It was commissioned for my practice by our ICB/ health board.
- Other, please specify: __________.

17. When did you first use an AI scribe in general practice?

[Dropdown month/ year with “I don’t know” option for month]

18. For what proportion of consultations do you currently use an AI scribe in general practice? Please estimate.

[slider 0-100%]

19. What did you use an AI scribe for?

[Open text box]

20. When did you discontinue the AI scribe(s) in general practice?

[Dropdown month/ year with “I don’t know” option for month]

21. Why did you discontinue using the AI scribe(s) in general practice? Please select all that apply.

- The AI scribe was no longer available.
- The cost of the AI scribe
- Medicolegal or liability issues regarding use of AI scribes
- AI scribes were not supported in my practice
- I did not think the AI scribe had any benefits.
- I had concerns around the use of an AI scribe in consultations.
- I saw negative media reports.
- Due to a cease letter from NHS England.
- Due to other official communication asking to cease using AI scribes.
- Other, please specify: __________.

22. Do you intend to use an AI scribe in general practice in the future?

- Yes
- No
- I don’t know

## Never user block

14. Which AI scribe(s) do you intend to use in general practice in the future? Please select all that apply.

- I do not intend to use an AI scribe.
- I don’t know yet.
- Abridge
- Accurx Scribe (Tandem Health)
- Ambience Healthcare
- Anima
- Anthem
- Augmedix Go
- BeamUp
- CLEARNotes
- DAX Copilot (Microsoft)
- DeepScribe
- Ezyscribe (Pennhealth Informatics)
- Freed AI
- HealthOrbit AI
- Heidi Health
- Innovaccer Provider Copilot
- i-Scribe
- Lexacom
- Lyrebird Health
- OmniMD AI Scribe
- Scribetech (Augnito Omni AI)
- Smart Notes AI dictation tool (The Access Group)
- Suki AI
- Sunoh.ai
- TORTUS
- TruBridge
- Twofold Health
- Vero Scribe
- Other, please specify: __________

## Benefits and risks (all participants)

23. Please indicate how much you agree with each of the following statements.

|  | **Strongly agree** | **Agree** | **Neutral** | **Disagree** | **Strongly disagree** |
| --- | --- | --- | --- | --- | --- |
| Using AI scribes in general practice has benefits for me as a GP. |  |  |  |  |  |
| Using AI scribes in general practice has benefits for patients. |  |  |  |  |  |
| My Using AI scribes in general practice has benefits for my practice. |  |  |  |  |  |
| Using AI scribes in general practice has benefits for the NHS. |  |  |  |  |  |

24. Do you perceive any benefits from using an AI scribe in general practice?

|  | **Yes, this is a benefit.** | **No, this is not a benefit.** | **I don’t know** |
| --- | --- | --- | --- |
| Having a real-time documentation of the patient consultation |  |  |  |
| Receiving standardised clinical notes of patient consultation |  |  |  |
| Automatic transfer of information into patient records |  |  |  |
| Automatic completion of referral letters or forms |  |  |  |
| Linking primary and secondary care documentation |  |  |  |
|  | **Yes, this is a benefit.** | **No, this is not a benefit.** | **I don’t know** |
| Reducing administrative burden |  |  |  |
| Enhancing accuracy of documentation |  |  |  |
| Reducing errors and omissions |  |  |  |
| Improving workflow |  |  |  |
| Potential for integration with clinical decision support |  |  |  |
| Reducing time spent writing notes |  |  |  |
|  | **Yes, this is a benefit.** | **No, this is not a benefit.** | **I don’t know** |
| Increasing job satisfaction |  |  |  |
| Reducing feelings of burnout |  |  |  |
| Alleviating cognitive load of remembering details to record later |  |  |  |
| Improving communication with patients during consultation |  |  |  |
| Patients may feel reassured by its use |  |  |  |

25. Do you perceive any additional *benefits* of an AI scribe in general practice?

[Open text box]

26. Who do you think could be affected by *problems or risks* of using an AI scribe in general practice?

|  | **Would be affected a lot** | **Would be affected a little** | **Would not be affected** |
| --- | --- | --- | --- |
| Me as a GP |  |  |  |
| Patients |  |  |  |
| My practice |  |  |  |
| The NHS |  |  |  |

27. Do you perceive any *problems or risks* from using an AI scribe in general practice?

|  | **Yes, this is a risk.** | **No, this is not a risk.** | **I don’t know** |
| --- | --- | --- | --- |
| Excessive length of notes |  |  |  |
| Time needed for extensive editing |  |  |  |
| Lack of AI scribe integration into the electronic health records |  |  |  |
| Inaccurate information, errors or misinterpretations in transcription or summary |  |  |  |
| Threats to patient safety due to inaccurate information |  |  |  |
| Concerns about safeguarding |  |  |  |
| Medicolegal threat due to errors or inaccuracies |  |  |  |
|  | **Yes, this is a risk.** | **No, this is not a risk.** | **I don’t know** |
| Risk of deskilling of GPs |  |  |  |
| Unclear data sources for training of AI scribes |  |  |  |
| Risk of discrimination from bias in training data |  |  |  |
| Data security risk of potentially identifiable patient data |  |  |  |
| Potential misuse of patient information |  |  |  |
| Conflicting guidance on whether and when to use an AI scribe in general practice |  |  |  |
| Lack of adequate evaluation of using an AI scribe in general practice |  |  |  |
| Unclear state of insurance or personal liability |  |  |  |
|  | **Yes, this is a risk.** | **No, this is not a risk.** | **I don’t know** |
| Patient’s medical issues are too complex to be captured adequately |  |  |  |
| Patient’s medical issues are too simple to warrant use of AI scribe |  |  |  |
| Negative impact on patient communication during consultation |  |  |  |
| Limited functionality for some accents, dialects, diverse speech patterns, and languages |  |  |  |
| Tacit signs and non-verbal communication not captured |  |  |  |
| Patient concerns about use of an AI scribe in general practice |  |  |  |
| Difficulties or time obtaining patient consent to use an AI scribe in general practice |  |  |  |
|  | **Yes, this is a risk.** | **No, this is not a risk.** | **I don’t know** |
| Inappropriate use of an AI scribe by other staff |  |  |  |
| Fear of being asked to see more patients due to time saved from using an AI scribe |  |  |  |
| Planetary impact of high energy usage of AI scribes |  |  |  |
| High costs using an AI scribe |  |  |  |
| Insufficient training in use of AI scribes |  |  |  |
| Initial implementation, system downtime or failure can disrupt workflows |  |  |  |

28. Do you perceive any additional *problems or* *risks* of an AI scribe in general practice?

[Open text box]

# Supplementary Table 1: Multivariable logistic regression analyses

### Table S1. Multivariable regression analysis on use of AI scribes (ever use vs never use)

|  | | **Ever users**  **n (%)** | **Never users**  **n (%)** | **Estimate** | **S.E.** | **p-value** | **OR** | **95% C.I. for Odds ratio** | |
| --- | --- | --- | --- | --- | --- | --- | --- | --- | --- |
|  |  |  |  |  |  |  |  | **Lower** | **Upper** |
| Sex | **Women** | 136 (56.4) | 105 (43.6) | 1 |  |  |  |  |  |
|  | Men | 234 (68.0) | 110 (32.0) | .42 | .20 | .036 | 1.52 | 1.03 | 2.25 |
| Ethnicity | **White** | 118 (59.6) | 80 (40.4) | 1 |  |  |  |  |  |
|  | Asian or Asian British | 125 (64.1) | 70 (35.9) | 0.33 | 0.24 | .172 | 1.39 | 0.87 | 2.24 |
|  | Black or Black British | 81 (68.1) | 38 (31.9) | 0.55 | 0.29 | .058 | 1.74 | 0.99 | 3.09 |
|  | Mixed or multiple ethnic groups | 11 (68.8) | 5 (31.3) | 0.64 | 0.65 | .324 | 1.90 | 0.56 | 7.61 |
|  | Other | 30 (63.8) | 17 (36.2) | 0.29 | 0.38 | .449 | 1.34 | 0.64 | 2.89 |
| Experience (in years) | **<5** | 156 (56.5) | 120 (43.5) | 1 |  |  |  |  |  |
|  | 6-15 | 146 (68.5) | 67 (31.5) | -0.03 | 0.26 | .922 | 0.98 | 0.59 | 1.61 |
|  | 16+ | 74 (67.9) | 35 (32.1) | 0.08 | 0.33 | .821 | 1.08 | 0.56 | 2.08 |
| Current role | **GP Partner** | 88 (69.8) | 38 (30.2) | 1 |  |  |  |  |  |
|  | Salaried GP | 168 (68.9) | 76 (31.1) | 0.34 | 0.30 | .249 | 1.41 | 0.79 | 2.52 |
|  | GP trainee | 73 (52.5) | 66 (47.5) | 0.16 | 0.37 | .656 | 1.18 | 0.58 | 2.42 |
|  | Locum GP | 43 (62.3) | 26 (37.7) | 0.19 | 0.38 | .620 | 1.21 | 0.57 | 2.59 |
|  | Other | 4 (20.0) | 16 (80.0) | -1.28 | 0.68 | .058 | 0.28 | 0.07 | 0.98 |
| Clinical sessions | **1-4** | 54 (45.4) | 65 (54.6) | 1 |  |  |  |  |  |
|  | 5-6 | 167 (70.2) | 71 (29.8) | 0.67 | 0.27 | .013 | 1.95 | 1.15 | 3.31 |
|  | 7+ | 155 (64.3) | 86 (35.7) | 0.62 | 0.28 | .024 | 1.86 | 1.08 | 3.19 |
| Private work | **No private work** | 207 (55.1) | 169 (44.9) | 1 |  |  |  |  |  |
|  | Occasional | 102 (75.0) | 34 (25) | 0.52 | 0.26 | .043 | 1.68 | 1.02 | 2.81 |
|  | Regular | 67 (77.9) | 19 (22.1) | 0.86 | 0.33 | .001 | 2.37 | 1.27 | 4.61 |
| GP trainer | **No** | 221 (55.0) | 181 (45.0) | 1 |  |  |  |  |  |
|  | Yes | 155 (79.1) | 41 (20.9) | 0.94 | 0.25 | <.001 | 2.55 | 1.56 | 4.25 |
| IT system | **EMIS Web** | 190 (60.9) | 122 (39.1) | 1 |  |  |  |  |  |
|  | SystmOne TTP | 163 (67.9) | 77 (32.1) | 0.50 | 0.21 | .015 | 1.65 | 1.10 | 2.48 |
|  | Other | 23 (50.0) | 23 (50.0) | 0.06 | 0.40 | .869 | 1.07 | 0.49 | 2.34 |
| Disability in local population | |  |  | -0.07 | 0.03 | .019 | 0.93 | 0.88 | 0.99 |

*Nagelkerke’s R^2^=* *.193*

# Supplementary Table 2: Perceived risks and benefits for stakeholders

### Table S2. Perceived benefits and risks for GPs, patients, practices, and the NHS

|  |  | **Current users (N=240)**  **N (%)** | **Past users (N=136)**  **N (%)** | **Never users (N=222)**  **N (%)** | **Pearson χ^2^ test** |
| --- | --- | --- | --- | --- | --- |
| **Using AI scribes in primary care has *benefits* for…*** | | | | |  |
| **GPs** | **Agree** | 230 (95.8) | 103 (75.7) | 153 (68.9) | χ^2^(4)=60.21, p<.001 |
|  | **Disagree** | 4 (1.7) | 4 (2.9) | 9 (4.1) |  |
| **Patients** | **Agree** | 202 (84.2) | 87 (64.0) | 143 (64.4) | χ^2^(4)=31.19, p<.001 |
|  | **Disagree** | 5 (2.1) | 6 (4.4) | 17 (7.7) |  |
| **GP practice** | **Agree** | 225 (93.8) | 101 (74.3) | 158 (71.2) | χ^2^(4)=44.18, p<.001 |
|  | **Disagree** | 4 (1.7) | 5 (3.7) | 8 (3.6) |  |
| **The NHS** | **Agree** | 218 (90.8) | 103 (75.7) | 161 (72.5) | χ^2^(4)=28.61, p<.001 |
|  | **Disagree** | 4 (1.7) | 6 (4.4) | 7 (3.2) |  |
| **Using AI scribes in primary care has *risks* for…*** | | | | |  |
| **GPs** | **Agree** | 122 (50.8) | 68 (50.0) | 137 (61.7) | χ^2^(4)=13.96, p=.007 |
|  | **Disagree** | 53 (22.1) | 23 (16.9) | 23 (10.4) |  |
| **Patients** | **Agree** | 96 (40.0) | 70 (51.5) | 123 (55.4) | χ^2^(4)=17.65, p=.001 |
|  | **Disagree** | 69 (28.8) | 22 (16.2) | 35 (15.8) |  |
| **GP practice** | **Agree** | 101 (42.1) | 64 (47.1) | 119 (53.6) | χ^2^(4)=11.55, p=.021 |
|  | **Disagree** | 71 (29.6) | 29 (21.3) | 38 (17.1) |  |
| **The NHS** | **Agree** | 104 (43.3) | 60 (44.1) | 117 (52.7) | χ^2^(4)=22.77, p<.001 |
|  | **Disagree** | 81 (33.8) | 32 (23.5) | 34 (15.3) |  |
| **A neutral answer option was also provided.* | | | | | |
